# An fMRI Study of the Influence of a History of Substance Abuse on Working Memory-Related Brain Activation in Schizophrenia

**DOI:** 10.3389/fpsyt.2014.00001

**Published:** 2014-01-21

**Authors:** Jessica A. Wojtalik, Deanna M. Barch

**Affiliations:** ^1^Department of Psychiatry, Washington University School of Medicine, St. Louis, MO, USA; ^2^Department of Psychology, Washington University in St. Louis, St. Louis, MO, USA; ^3^Edward Mallinckrodt Institute of Radiology, Washington University School of Medicine, St. Louis, MO, USA

**Keywords:** schizophrenia, substance abuse, *n*-back, working memory, fMRI, neurocognition

## Abstract

There has been little investigation of the effects of past substance abuse (SA) on working memory (WM) impairments in schizophrenia. This study examined the behavioral and neurobiological impact of past SA (6 months or longer abstinence period) on WM in schizophrenia. Thirty-seven schizophrenia patients (17 with past SA and 20 without) and 32 controls (12 with past SA and 20 without) completed two versions of a two-back WM task during fMRI scanning on separate days. Analyses focused on regions whose patterns of activation replicated across both *n*-back tasks. Schizophrenia patients were significantly less accurate than controls on both *n*-back tasks. No main effects or interactions with past SA on WM performance were observed. However, several fronto-parietal-thalamic regions showed an interaction between diagnostic group and past SA. These regions were significantly more active in controls with past SA compared to controls without past SA. Schizophrenia patients with or without past SA either showed no significant differences, or patients with past SA showed somewhat less activation compared to patients without past SA during WM. These results suggest robust effects of past SA on WM brain functioning in controls, but less impact of past SA in schizophrenia. This is consistent with previous literature indicating less impaired neurocognition in schizophrenia with SA.

## Introduction

The lifetime prevalence rate of substance abuse (SA) among patients diagnosed with schizophrenia is approximately 50% (1). Despite the extent of SA in schizophrenia, neurocognitive (2, 3) and neuroimaging (4) evidence of the impact of past SA in this illness has been relatively inconsistent. These studies have primarily focused on patients either currently using substances or recently abstinent from SA. While understanding acute effects are important, it is also critical to determine whether past SA has any lasting effects on neurocognitive and/or neurobiological functioning in schizophrenia. Examining the impact of past SA on working memory (WM) in schizophrenia patients is vital, given the evidence for long-term WM impairments persisting after long-term abstinence from SA in non-psychosis samples (5–14). It is also well known that schizophrenia patients are burdened with robust deficits in WM performance (15) and brain functioning (16). Therefore, investigation of possible residual effects of past SA on WM impairments could provide further understanding of the complex interaction between neurocognitive impairment and SA in schizophrenia, and could have implications for prevention and intervention in cognitive impairments in schizophrenia.

Several studies have shown a negative impact of past SA on WM brain functioning in non-psychosis samples. After 28 days of abstinence, adolescent marijuana users displayed aberrant brain activations evoked during WM in the dorsolateral prefrontal cortex (DLPFC), parietal cortex, and occipital regions compared to non-abusers (17). Altered WM-related activation patterns in frontal–parietal and cerebellar regions have been observed in males with prior alcoholism compared to healthy males (18, 19). Tomasi and colleagues (20) revealed residual negative effects of cocaine on brain functioning in dopaminergic-regulated brain regions in abstinent cocaine abusers while engaged in WM. While these aforementioned studies did not focus on schizophrenia-spectrum comorbidity, their results indicate functional WM impairments associated with past SA, even after long-term abstinence.

Despite the aforementioned neurobiological evidence for residual WM deficits associated with past SA in non-psychosis samples, relatively few research studies exist examining these effects in schizophrenia. The current literature has mixed results that are somewhat difficult to interpret (21). Starting with the behavioral performance literature, an additional SA diagnosis in schizophrenia did not exacerbate WM impairments compared to patients without past SA (22). Rodriguez-Jimenez and others (23) also found no differences in executive function between schizophrenia patients with and without past SA; although the predictors of poorer executive function did differ. Past cannabis abuse has been generally associated with similar or better neurocognition in patients with schizophrenia compared to patients without past cannabis abuse (2, 3, 24, 25). A history of alcoholism in schizophrenia has also been shown to not convey an additional burden on neurocognitive impairments (26). However, Allen and colleagues (27) observed an association between past alcohol abuse and cognitive–perceptual neurologic dysfunction in schizophrenia patients. Past and current cocaine dependent schizophrenia patients have been shown to not significantly differ in neurocognitive deficits (28). However, other work has shown that past cocaine abuse and dependence exacerbated memory and learning impairments in schizophrenia with and without past cocaine use (29, 30).

An interesting differential effect of past SA on neurocognitive functioning in schizophrenia patients compared to controls has been recently observed. Jockers-Scherübl and colleagues (31) found that controls with past cannabis abuse displayed decreased neurocognitive functioning compared to controls without past cannabis abuse. Conversely, schizophrenia patients with past cannabis abuse had slightly better neurocognitive functioning compared to patients without past cannabis abuse. Donoghue and others (32) also found first-episode schizophrenia patients with and without past SA performed similarly on neurocognitive assessments, while the controls with past SA performed more poorly on measures of WM and executive function compared to controls without past SA. Taken together, clear inferences are difficult to make regarding the impact of past SA (short or long-term) on neurocognitive functioning in schizophrenia. Further, two recent meta-analyses concluded that these mixed findings may be accounted for by heterogeneous variables in schizophrenia samples studied such as age, type of SA, and status of abuse (2, 3).

There are also fairly limited and mixed findings with regard to the effects of past SA on indices of brain structure and function in schizophrenia. To our knowledge, there have been no fMRI investigations of SA-related WM deficits in schizophrenia. Two social–cognitive fMRI studies indicated that schizophrenia patients with past SA had relatively preserved medial prefrontal cortex activity during processing of social emotion (33) and affect regulation (34) compared to patients without past SA. Similar to the differential impact of past SA on neurocognition in schizophrenia and controls discussed above, Rentzsch and colleagues (35) observed a P50 sensory gating deficit, measured by EEG, only in the controls with past cannabis abuse compared to controls without past cannabis abuse. Schizophrenia patients with and without past cannabis abuse did not significantly differ in P50 sensory gating. In contrast, Thoma and colleagues (36) found that schizophrenia patients with a history of alcohol abuse had the greatest deficits in WM and P50 sensory gating compared to schizophrenia patients without past alcohol abuse and controls with and without past alcohol abuse.

A review of the SA neuroimaging literature by Potvin and colleagues (4) suggested some evidence for exacerbated structural abnormalities associated with alcohol and cannabis use in schizophrenia. In a voxel-based morphometry investigation, schizophrenia patients with past SA were found to have gray matter volume decreases in the anterior cingulate and frontopolar areas associated with impulsivity compared to non-abusing schizophrenia (37). Additionally, relative to patients without past SA, previous exposure to cannabis (38, 39) and alcohol (40–42) has been associated with worsened structural abnormalities in cingulate, subcortical (e.g., hippocampus, striatum, and thalamus), and cerebellar regions in schizophrenia patients with past SA. In contrast, other studies have demonstrated that schizophrenia patients with past SA have qualitatively less structural MRI anomalies compared to patients without past SA (43).

As described above, there is limited and mixed evidence regarding the impact of past SA on WM functioning in schizophrenia. Thus, the aim of this investigation was to do an initial examination of the impact of past SA on brain activation during WM performance in schizophrenia patients with and without past SA compared to healthy controls with and without past SA. To increase confidence in our findings through replication, participants completed one version of a two-back WM task during one scanning session and then completed a second version of a two-back WM task during a second scanning session on a different day. Given the inconsistent results in the prior literature, it is difficult to make specific predictions about the behavioral and functional impairments associated with 6 months or longer abstinence periods in the schizophrenia patients. However, the literature suggesting detrimental effects of past SA on WM performance and brain activation in non-psychosis samples leads us to hypothesize that schizophrenia patients with past SA will display the greatest degree of WM impairments in performance and brain activation in regions implicated in WM impairment, such as the DLPFC (44) and other frontal and cingulate regions (6).

## Materials and Methods

### Participants

Participants were recruited through the clinical core of the Conte Center for the Neuroscience of Mental Disorders (CCNMD) at Washington University in St. Louis. Exclusion criteria included: (a) any type of substance abuse/dependence present within the past 6 months; (b) the presence of any clinically unstable or severe medical disorder; (c) current or past head injury with documented neurological sequelae, and/or causing loss of consciousness; (d) meeting DSM-IV criteria for mental retardation; and (e) pregnancy, or any contraindication to MR. Controls were excluded if they had any lifetime history of, or first-order family member with, an Axis I psychotic disorder. Controls were also excluded if they endorsed any personal current or past mood or anxiety disorder other than specific phobias.

A total of 37 participants with DSM-IV-TR schizophrenia (17 with past SA and 20 without) and 32 controls (12 with past SA and 20 without) completed the *n*-back version A task. Thirty-one of the above schizophrenia participants (15 with past SA and 16 without) and 29 control participants (17 with past SA and 12 without) also completed the *n*-back version B task on a different scanning day. Fewer subjects completed the *n*-back version B task because they failed to return to the second scanning session on a different day. All participants provided written informed consent in accordance with Washington University Human Subjects Committee’s criteria. Schizophrenia diagnoses were based on a Structured Clinical Interview for DSM-IV-TR (45) performed by a trained masters-level clinician. The clinician also had access to past and present medical records and to corroborative personal sources (e.g., family), and combined all data to arrive at a diagnosis. This rater regularly participated in clinical interview and reliability training sessions as part of the CCNMD. Clinical symptoms were rated using the Scale for the Assessment of Positive Symptoms [SAPS; (46)] and Negative Symptoms [SANS; (46)]. Based on previous factor analytic studies of the SAPS/SANS (47), we created the following symptom domain scores: (1) positive symptoms – hallucinations and delusions; (2) negative symptoms – alogia, anhedonia, avolition, affective flattening, and attentional impairment; and (3) disorganization – bizarre behavior, positive thought disorder, and inappropriate affect. All individuals with schizophrenia were taking antipsychotic medications at the time of participation in the study. For both control and schizophrenia participants, the Structured Clinical Interview for the DSM-IV-TR (45) was also used to determine criteria for lifetime substance abuse/dependence diagnoses and rule-out any current SA. Past SA was defined as a period longer than 6 months without any SA or complications from abuse, determined by self-report during the DSM-IV-TR diagnostic interview and other reliable clinical sources (e.g., psychiatric records and/or personal references).

### Tasks and materials

#### *n*-back version A

The first WM task was a two-back version of an *n*-back using either words or faces (unfamiliar). The two-back version was used because of previous literature indicating this level of WM processing elicits peak activation in schizophrenia patients compared to healthy controls (48). We did not use a three-back level because of concerns that it would exceed capacity limits for many patients and thus would not provide valid estimates of WM-related brain activation in patients. We could have also used a one-back condition, but were concerned that this would not be sufficiently taxing. There were three runs with words and three runs with faces. The words were concrete English nouns one to four syllables in length. The unfamiliar faces were neutral in emotion and included both male and female genders. These same stimuli have been used in a number of previous studies (49–52). Prior to each run, participants watched a video that was either neutral (once for words and once for faces), pleasant (once for words and once for faces), or unpleasant (once for words and once for faces), with the order counterbalanced across subjects. The videos were identical to those used in previous investigations (53), and consisted of 9–10 min digitally presented (QuickTime format, resolution 320 × 240) video clips from comedy, documentary, and horror movie genres (54). Headphones were utilized to enable participants to hear video-audio and dampen scanner noise. The effect of the videos on performance was not of interest in the current study, and therefore all analyses were collapsed across video conditions. In each run, participants were instructed to press the target button if the current stimulus on the screen matched the stimulus previously seen two trials back and to press the non-target button for any other stimuli presented on the screen. The sequence of block events for this task included four initial fixation trials to allow the signal to reach a steady-state, followed by 4 blocks of 16 two-back task trials (2.5 s stimulus presentations, 500 ms inter-stimulus delays) interleaved with 4 blocks of 10 fixation trials displaying a continuous cross-hair.

#### *n*-back version B

For the second scanning session on a different day, participants performed six additional runs of a two-back task, three with words and three with unfamiliar faces. For this version of the *n*-back, the unfamiliar faces were displaying either happy, fearful, or neutral expressions (55, 56) and the word stimuli were neutral, positive, or negative. The face stimuli matched in lighting, location, distance, and exposure. Participants were provided the same instructions as in the first two-back task described above. The sequence of block events for this task included four initial fixation trials to allow the signal to reach a steady-state, followed by 3 blocks of 32 two-back task trials (2.5 s stimulus presentations, 500 ms inter-stimulus delays) interleaved with 4 blocks of 16 fixation trials displaying a continuous cross-hair. Task blocks either included all neutral stimuli, 16 neutral and 16 positive stimuli, or 16 neutral and 16 negative stimuli. As with the video task, the influence of the emotional valence of the stimuli on performance was not of interest in the current study, and all analyses were collapsed across emotion type. Visual stimuli were generated by a G3 Macintosh computer and PsyScope, and projected onto a computer screen behind the subject’s head within the imaging chamber. Participants saw the screen through a mirror positioned approximately 8 cm above their face.

### fMRI image acquisition and processing

Functional scanning was performed on a 3-T Siemens Allegra head-dedicated system at the Research Imaging Center of the Mallinckrodt Institute of Radiology at the Washington University Medical School. First, a low resolution 3D sagittal T1-weighted MP-RAGE acquisition image was obtained (TE = 2.9 ms, TR = 6.6 ms, flip angle = 8°, 96 × 128 acquisition matrix, 1 acquisition, 80 slices, 2 mm × 2.67 mm × 2 mm voxels). This MP-RAGE was then warped to Talairach space (57). Second, a T2 image was subsequently acquired in the same position as the blood-oxygenation-level-dependent (BOLD) images (TE = 96 ms, TR = 5 s, 189 × 256 acquisition matrix, 48 slices, 1.02 mm × 1 mm × 3 mm voxels), and used as a bridge to facilitate the registration of the T1-weighted images acquired during the structural imaging session and the functional (T2*-weighted) images. The slice locations for functional images were placed based on the results of the computerized slice pre-registration. The functional images were collected in runs using an asymmetric spin-echo echo-planar sequence sensitive to BOLD contrast (T2*) (TR = 3000 ms, TE = 25 ms, FOV = 205 mm, flip = 90°). During each functional run, sets of 32 contiguous axial images with isotropic voxels (4 mm^3^) were acquired parallel to the anterior–posterior commissure plane.

MR data were reconstructed into images, and normalized across runs by scaling whole-brain signal intensity to a fixed value (mode of 1000), and removing the linear slope on a voxel-by-voxel basis to counteract effects of drift. To correct for head motion, the images were aligned using six parameter rigid-body rotation and translation correction algorithms (58, 59). The images were then registered to a common Talairach atlas space (57) using 12 parameter linear (affine) transformations of the participant’s average MP-RAGE structural image warped into T2 space, and then using the T2 image to register the T2* and T1 images. Finally, the normalized, registered fMRI images were spatially smoothed with a 9-mm FWHM Gaussian kernel.

### Statistical analysis

#### Behavioral data

To investigate the effects of past SA on WM accuracy performance in schizophrenia patients and controls on both versions A and B of the *n*-back, we used a repeated measures design implementing a General Linear Model (GLM) executed in SPSSv19. These analyses included stimulus type (words vs. faces) as a within subject factor and diagnostic group (schizophrenia vs. controls) and past SA (no vs. yes) as between-subject factors, with mean WM accuracy as the dependent variable.

#### fMRI data

For the functional imaging data, GLMs were modeled within each participant to estimate voxel-wise magnitudes of task-related activity, with one GLM for each of the two *n*-back tasks. Each GLM included regressors for linear trends within runs and baseline shifts between runs computed for every subject. BOLD responses to each block-type (e.g., words and faces) were modeled as “boxcar” functions, and then convolved with a canonical hemodynamic response function to generate separate estimates for each condition. For clarity and brevity, only the main effects and interactions involving past SA (e.g., main effects of past SA and diagnostic group, and the past SA × diagnostic group interaction) were considered when examining differences in activations during each WM task independently. Using an in-house software program (FIDL), estimated GLMs from each individual participant were then entered into whole-brain voxel-wise mixed effects analyses of variance (ANOVA) using stimulus type (words vs. faces) as the within subject factor, diagnostic group (control vs. schizophrenia) and past SA (no vs. yes) as between-subject factors, and subjects as a random factor.

Because our goal was to examine brain regions that showed consistent associations with past SA, we focused on identifying brain regions that showed the same effects across both versions A and B of the two-back tasks. Replicating across WM functioning between the two tasks was also done to increase confidence in our findings of WM-related brain activation and to weaken any effects specific to either the *n*-back version A or the *n*-back version B. To do so, we thresholded the whole-brain statistical maps for the *n*-back version A and the *n*-back version B at *Z* > 2.5 and *k* = 13 based on Monte Carlo simulations, and created conjunction maps of regions showing the same effects across both tasks. Monte Carlo simulations were utilized to protect against inflated type 1 error (60, 61). This combination provides *p* < 0.05 whole-brain false positive protection for inferences regarding brain regions showing effects across both tasks. We then used planned contrasts in these regions to clarify the direction of effects and to address the influence of any potential confounding variables that may covary with past SA.

## Results

### Demographic characteristics

Overall, the schizophrenia patients and controls did not significantly differ in age, gender, ethnicity, handedness, or highest parental education. However, the mean number of years of education was higher in the control group, and the rate of smoking was higher for the schizophrenia group. With regard to subgroup differences (see Table 1), controls with past SA were significantly older than the controls without past SA (*t* = −2.58, *p* = 0.015). In addition, controls without past SA had parents with significantly higher levels of education compared to the parents of controls with past SA (*t* = 2.85, *p* = 0.008). Subsequently, age and highest parental education were entered into secondary behavioral and fMRI analyses as confounding variables. No significant differences emerged between the schizophrenia patients with and without past SA, with the exception of the schizophrenia patients with past SA having slightly higher disorganized symptomology (*t* = −2.06, *p* = 0.046). Past SA was not correlated with neither positive symptoms (*r* = 0.24, *p* = 0.145) nor negative symptoms (*r* = 0.01, *p* = 0.970) in the schizophrenia patients. Specific past SA characteristics for the subgroups of control and schizophrenia participants are presented in Table 2.

**Table 1:** **Demographic characteristics and clinical characteristics of participants**.

Characteristic	Schizophrenia				Controls			
	SCZ (*n* = 20)	SCZ + SA (*n* = 17)	*t* or χ^2^	*p*	CON (*n* = 20)	CON + SA (*n* = 12)	t or χ^2^	*p*
Age (years)	35.85 (8.28)	38.76 (10.23)	−0.95	0.345	32.65 (10.67)	42.08 (8.67)	−2.58	0.015
Gender (% male)	11 (55%)	14 (82%)	3.13	0.094	13 (65%)	8 (67%)	0.01	1.000
Ethnicity (%)			0.23	0.746			0.07	1.000
African American	11 (55%)	8 (47%)			9 (45%)	6 (50%)		
Caucasian	9 (45%)	9 (53%)			11 (55%)	6 (50%)		
Handedness	62.50 (57.15)	51.47 (58.54)	0.57	0.567	76.50 (31.54)	62.08 (56.10)	0.81	0.427
Education (years)	13.10 (2.70)	13.00 (1.96)	0.12	0.900	16.22 (4.78)	14.70 (3.09)	0.97	0.335
Parental education (years)	13.66 (3.56)	14.42 (2.62)	−0.67	0.508	13.85 (2.32)	11.66 (1.61)	2.85	0.008
Smoker (% yes)	9 (45%)	13 (76%)	3.77	0.092	2 (10%)	3 (25%)	1.28	0.338
Positive symptoms rating	1.25 (1.37)	1.91 (1.31)	−1.49	0.144	0.00 (0.00)	0.04 (0.15)	−1.00	0.341
Negative symptom rating	1.85 (1.03)	1.83 (0.78)	0.03	0.970	0.25 (0.36)	0.27 (0.20)	−0.19	0.850
Disorganization symptoms rating	0.81 (0.72)	1.33 (0.79)	−2.06	0.046	0.38 (0.36)	0.60 (0.59)	−1.13	0.275
Age of onset (years)	18.30 (7.45)	19.88 (7.95)	−0.62	0.537	–	–	–	–
Antipsychotic mediations			1.80	0.406	–	–	–	–
Atypical	16 (80%)	15 (88%)						
Typical	2 (10%)	2 (12%)						
Combination atypical/typical	2 (10%)	0 (0%)						

*SCZ, schizophrenia patients; SCZ + SA, schizophrenia patients with past substance abuse; CON, control participants; CON + SA, control participants with past substance abuse*.

**Table 2:** **Past substance use diagnostic characteristics of the schizophrenia and control participants**.

Substance use characteristics	SCZ + SA (*n* = 17)	CON + SA (*n* = 12)	*t* or χ*^2^*	*p*
	*N* (%)	*N* (%)	
Alcohol only diagnosis	–	5 (41.7%)	8.55	0.007
Alcohol	11 (64.7%)	9 (75%)	0.34	0.694
Cannabis	11 (64.7%)	4 (33.3%)	2.77	0.139
Cocaine	3 (17.6%)	4 (33.3%)	0.94	0.403
Hallucinogen	3 (17.6%)	1 (8.3%)	0.51	0.622
Stimulant	2 (11.8%)	0	1.51	0.498
Opiate	1 (5.9%)	0	0.73	1.000
Sedative	2 (11.8%)	0	1.51	0.498
Polysubstance	3 (17.6%)	1 (8.3%)	0.51	0.622
Number of dependence diagnoses	15 (88.2%)	7 (58.3%)	3.43	0.092
More than one substance use diagnosis	14 (82.4%)	5 (41.7)	5.15	0.046
Number of substance use diagnoses	–	–	5.85	0.211
1 Diagnosis	3 (17.6)	7 (58.3)	–	–
2 Diagnoses	8 (47.1)	3 (25)	–	–
3 Diagnoses	4 (23.5)	2 (16.7)	–	–
4 Diagnoses	1 (5.9)	0	–	–
5 Diagnoses	1 (5.9)	0	–	–

*SCZ + SA, schizophrenia patients with past substance abuse; CON + SA, control participants with past substance abuse. Alcohol through polysubstance includes both abuse and dependence diagnoses*.

### Working memory accuracy

A significant main effect of diagnostic group for the *n*-back version A (*F* = 11.45, *p* = 0.001) and *n*-back version B (*F* = 13.94, *p* = < 0.001) was observed, such that schizophrenia patients were significantly less accurate than controls (see Table 3). There was, however, no significant main effect of past SA for either the *n*-back version A (*F* = 0.92, *p* = 0.340) or *n*-back version B (*F* = 0.58, *p* = 0.446), nor was there a significant diagnostic group by past SA interaction for either the *n*-back version A (*F* = 0.01, *p* = 0.894) or *n*-back version B (*F* = 0.06, *p* = 0.807).

**Table 3:** **Accuracy during the working memory tasks**.

Group	*n*-Back version A		*n*-Back version B	
	Word	Face	Word	Face
	Mean (SD)	Mean (SD)	Mean (SD)	Mean (SD)
CON	0.91 (0.08)	0.93 (0.06)	0.97 (0.02)	0.93 (0.04)
CON + SA	0.91 (0.09)	0.91 (0.08)	0.95 (0.03)	0.92 (0.05)
SCZ	0.88 (0.07)	0.86 (0.08)	0.91 (0.07)	0.85 (0.14)
SCZ + SA	0.85 (0.05)	0.84 (0.08)	0.90 (0.06)	0.84 (0.06)

*SCZ, schizophrenia patients; SCZ + SA, schizophrenia patients with past substance abuse; CON, control participants; CON + SA, control participants with past substance abuse*.

### fMRI results

As described above, we used voxel-wise mixed effects ANOVAs with diagnostic group and past SA as between-subjects factors and stimulus type as a within subject factor to analyze differences in activation that replicated across both versions A and B of the *n*-back. We focused on regions that displayed significant main effects or interactions with group and/or past SA. First, as shown in Table 4, several regions engaged during both versions of the *n*-back demonstrated a significant main effect of diagnostic group. When compared to the control participants, schizophrenia patients displayed significantly greater WM-related activation in the left superior temporal gyrus, left superior parietal lobule, left cerebellum tonsil and pyramis, and the right supramarginal gyrus. Second, there were a few regions that demonstrated a significant main effect of a past SA (see Table 4). During the *n*-back version A and *n*-back version B, the participants with past SA displayed increased activation in the left superior temporal gyrus, left middle temporal gyrus, and the left angular gyrus while participants without past SA were deactivating these same regions. Participants with past SA also deactivated the left posterior cingulate gyrus less than participants without past SA.

**Table 4:** **Brain regions showing main effects of diagnostic group and past substance abuse during both versions of the *n*-back**.

Region	BA	*x*	*y*	*z*	*z*-Value		Activity pattern	
					*n*-Back version A	*n*-Back version B	*n*-Back version A	*n*-Back version B
**MAIN EFFECT OF DIAGNOSTIC GROUP (CONTROL VS. SCHIZOPHRENIA)**								
L superior temporal gyrus	22	−64	−32	13	2.92	3.66	CON(−) < SCZ	CON(−) < SCZ
L superior parietal lobule	7	−32	−46	62	3.15	3.43	CON < SCZ	CON < SCZ
L cerebellum tonsil	−	−33	−48	−34	2.98	3.63	CON < SCZ	CON < SCZ
L cerebellum pyramis	−	−43	−71	−31	3.26	3.57	CON < SCZ	CON < SCZ
R supramarginal gyrus	40	63	−38	29	2.85	2.92	CON(−) < SCZ	CON(−) < SCZ
**MAIN EFFECT OF PAST SUBSTANCE ABUSE (NO VS. YES)**								
L superior temporal gyrus	22	−63	−17	2	2.73	2.96	NSA(−) < SA	NSA(−) < SA
L middle temporal gyrus	21	−65	−37	−12	3.04	3.71	NSA(−) < SA	NSA(−) < SA
L angular gyrus	39	−49	−61	38	2.80	2.91	NSA(−) < SA	NSA(−) < SA
L posterior cingulate	31	−7	−44	38	2.64	3.28	NSA(−) > SA(−)	NSA(−) > SA(−)

*Activity patterns are activations, except when denoted with (−), indicating deactivation. Coordinates (*x, y, z*) reflect Talairach template space (57). BA, Brodmann area; L, left; R, right; CON, all control participants; SCZ, all schizophrenia patients; NSA, all participants without past substance abuse; SA, all participants with past substance abuse*.

**Figure 1:**
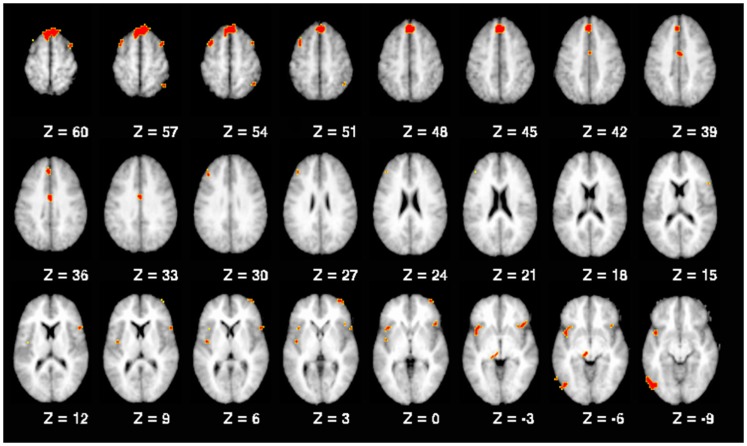
**Working memory-related brain regions demonstrating a significant interaction between diagnostic group and past substance abuse**. Note: all regions replicated across both *n*-back tasks, as described in the text. Brain slices are shown with the right hemisphere on the right side.

**Figure 2:**
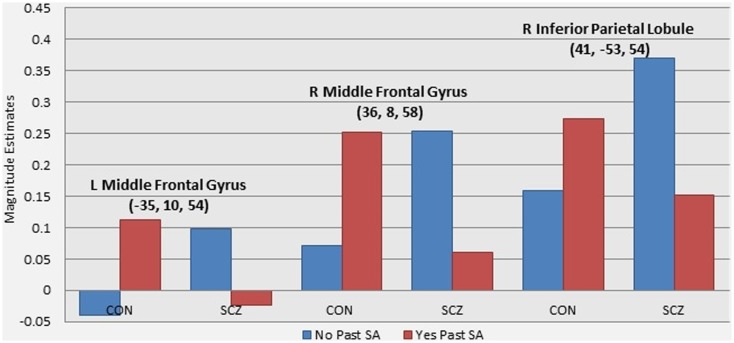
**Graphs illustrating the differential effect of past substance abuse across groups in WM-related brain activity**. Note: these graphs present magnitude estimates from follow up analyses of the patterns of activation driving the interaction effect of diagnostic group and presence of past substance abuse. The three regions presented were the only regions to show significant differences between schizophrenia patients with and without past substance abuse (see Table 4).

A number of regions showed significant activation differences across both versions of the *n*-back tasks as a function of the interaction of diagnostic group and past SA (see Figure 1; Table 5). Subsequent analysis with planned contrasts to follow up on the sources of these interactions revealed that they largely reflected robust differences across both versions of the *n*-back tasks between controls with and without past SA (see Table 5). These regions included the left superior frontal gyrus, left DLPFC, left fusiform, left insula, left anterior/middle cingulate cortex, left thalamus, right orbitofrontal gyrus, right precentral gyrus, right inferior parietal lobule, and the bilateral middle frontal gyrus. With the exception of the right inferior parietal lobule during the *n*-back version A, these fronto-partial-thalamic regions activated significantly more in the controls with past SA relative to controls without past SA. Also seen in Table 5, relatively few regions displayed significant differences between the schizophrenia patients with and without past SA (e.g., bilateral middle frontal gyrus and the right interior parietal lobule). During only the *n*-back version A, the schizophrenia patients with past SA demonstrated significantly less activation in these three regions compared to patients without past SA (see Figure 2). All the aforementioned main effects and interaction results remained significant in secondary analyses when age and highest parental education were included as confounding variables. Thus, results presented throughout were from the original analyses not covarying for these variables.

**Table 5:** **Brain regions displaying a significant interaction between diagnostic group and past substance abuse during both versions of the *n*-back**.

Region	BA	*x*	*y*	*z*	*z*-Value		Activity pattern			
					*n*-Back version A	*n*-Back version B	*n*-Back version A		*n*-Back version B	
**GROUP (CONTROL VS. SCHIZOPHRENIA) ***×*** PAST SUBSTANCE ABUSE (NO VS. YES)**										
L superior frontal gyrus	8	0	29	53	3.54	4.48	C(−) < CSA	S = SSA	C(−) < CSA	S = SSA
L DLPFC	9	−40	31	27	2.75	3.21	C < CSA	S = SSA	C < CSA	S = SSA
L middle frontal gyrus	6	−35	10	54	3.26	2.84	C(−) < CSA	S > SSA(−)	C(−) < CSA	S = SSA
L fusiform gyrus	19	−49	−72	−10	3.04	3.27	C < CSA	S = SSA	C < CSA	S = SSA
L insula	13	−40	9	−3	3.03	3.34	C < CSA	S = SSA	C < CSA	S = SSA
L insula	13	−41	−7	5	2.80	2.67	C(−) < CSA	S = SSA	C < CSA	S = SSA
L ant./mid. cingulate	24	0	-6	36	3.71	3.03	C(−) < CSA	S = SSA(−)	C(−) < CSA	S = SSA
L thalamus	−	−9	−28	−4	2.57	2.77	C < CSA	S = SSA	C < CSA	S = SSA
R middle frontal gyrus	10	36	55	3	2.73	3.03	C(−) < CSA	S = SSA	C(−) < CSA	S = SSA
R middle frontal gyrus	6	36	8	58	3.20	2.97	C < CSA	S > SSA	C < CSA	S = SSA
R orbitofrontal gyrus	47	41	18	−2	2.68	3.60	C < CSA	S = SSA	C < CSA	S = SSA
R precentral gyrus	44	53	13	8	2.82	3.17	C < CSA	S = SSA	C < CSA	S = SSA
R inferior parietal lobule	40	41	−53	54	2.63	3.14	C = CSA	S > SSA	C < CSA	S = SSA

*Activity patterns are activations, except when denoted with (−), indicating deactivation. Coordinates (*x, y, z*) reflect Talairach template space (57). “ =”, represents no significant difference in *post hoc* analysis; BA, Brodmann area; L, left; R, right; DLPFC, dorsolateral prefrontal cortex; ant., anterior; mid., middle; C, control participants without past substance abuse; CSA, control participants with past substance abuse; S, schizophrenia patients without past substance abuse; SSA, schizophrenia patients with past substance abuse*.

### *Post hoc* analyses comparing schizophrenia and controls without past SA

A number of regions showing interactions between diagnostic group and past SA in this study are those who have been similarly identified as showing group differences during WM and executive function tasks in prior studies (62). Thus, we wanted to examine if these regions would show diagnostic group differences if we compared the schizophrenia patients without past SA to the controls without past SA (the typical sample composition in imaging studies within schizophrenia). As shown in Table 6, most of these regions showed significant group differences across both *n*-back tasks, with the exception of the DLPFC. The schizophrenia patients without past SA displayed significantly greater activation compared to controls without past SA. Although a few regions were no longer significantly different (e.g., bilateral middle frontal gyrus and right inferior parietal lobule), most regions remained significantly different from controls when patients with past SA were added to the sample of patients without past SA. Such results suggest that a differential history of past SA in schizophrenia samples is not responsible for group differences from controls in functional brain activity during WM tasks.

**Table 6:** ***Post hoc* analyses examining the source of interaction effects between diagnostic group and past substance abuse**.

Region	BA	*x*	*y*	*z*	Activity pattern			
					CON (no past SA) vs. SCZ (no past SA)		CON (no past SA) vs. SCZ (with and without past SA)	
					*n*-Back version A	*n*-Back version B	*n*-Back version A	*n*-Back version B
L superior frontal gyrus	8	0	29	53	C < S***	C < S***	C < S+**	C < S+***
L DLPFC	9	−40	31	27	C = S	C = S	C = S+	C = S+
L middle frontal gyrus	6	−35	10	54	C < S**	C < S*	C = S+	C = S+
L fusiform gyrus	19	−49	−72	−10	C < S**	C < S*	C < S+**	C = S+
L insula	13	−40	9	−3	C < S**	C < S**	C < S+**	C < S+**
L insula	13	−41	−7	5	C < S*	C < S*	C < S+**	C < S+***
L ant./mid. cingulate	24	0	−6	36	C < S**	C < S***	C < S+***	C < S+***
L thalamus	−	−9	−28	−4	C < S***	C < S*	C < S+**	C < S+*
R middle frontal gyrus	10	36	55	3	C < S**	C < S***	C < S+**	C < S+*
R middle frontal gyrus	6	36	8	58	C < S**	C < S*	C = S+	C = S+
R orbitofrontal gyrus	47	41	18	−2	C < S*	C < S**	C = S+	C < S+**
R precentral gyrus	44	53	13	8	C < S*	C < S*	C < S+*	C < S+***
R inferior parietal lobule	40	41	−53	54	C < S*	C < S*	C = S+	C = S+

Results are from two-sample *t*-tests. Coordinates (*x, y, z*) reflect Talairach template space (57). “ =,” represents no significant difference; BA, Brodmann area; L, left; R, right; DLPFC, dorsolateral prefrontal cortex; ant., anterior; mid., middle; C, control participants without past substance abuse; S, schizophrenia patients without past substance abuse; S+, combined schizophrenia patients with and without past substance abuse; *p < 0.05, **p < 0.01, ***p < 0.001.

## Discussion

The goal of this initial investigation was to examine the effects of past SA in schizophrenia patients compared to healthy controls. This was accomplished by assessing both behavioral and neural activity that replicated across two versions of a two-back WM task in schizophrenia patients and controls with and without past SA. Although we had predicted that schizophrenia patients with past SA would demonstrate the highest level of WM impairment, our results indicated that past SA did not exacerbate behavioral or functional WM impairment in schizophrenia. An interesting differential effect of past SA was observed between controls and schizophrenia patients in WM-related brain functioning. Specifically, a number of significant WM-related brain activation differences were observed between controls with and without past SA, while only a few regions showed significantly different task-related activation between schizophrenia patients with and without past SA. These results remained significant after age and highest parental education were considered as cofounding variables. The results are discussed in more detail below.

As expected, the schizophrenia patients were significantly less accurate than controls on both versions of the *n*-back. However, no significant differences in WM accuracy were observed in either controls or schizophrenia patients with and without past SA. These findings are consistent with other previous investigations observing equivalent or better neurocognitive performance in abstinent schizophrenia patients with past SA compared to patients without past SA (25, 26, 63). In particular, a WM and multi-tasking investigation reported by Thoma and Daum (22) indicated no additive impact of past SA on WM deficits in schizophrenia patients. There have been a few studies, however, of past SA in schizophrenia that have shown exacerbated neurocognitive impairment (29, 30, 64). These differences may be accounted for by the individual effects of the SA (29, 30, 64), and/or inpatient samples with more severe neurocognitive impairments (64). Regarding the behavioral findings in the controls, other fMRI investigations of WM have also reported comparable performances between controls with and without past SA (17, 19, 65).

Differential effects of past SA on WM-related brain functioning were demonstrated between control and schizophrenia groups in regions that showed a significant interaction with diagnostic group and past SA. Subsequent investigation of these interaction patterns indicated that past SA had the greatest impact in the control group, with controls with past SA showing significant increased activation compared to controls without past SA in a range of fronto-parietal-thalamic regions that replicated across both versions of the *n*-back. In contrast, the schizophrenia patients with and without past SA only differed significantly in decreased WM-related activation in the bilateral middle frontal gyrus and the right inferior parietal lobule, only observed for version A of the *n*-back.

Our findings in the controls are consistent with previous studies showing residual SA-related abnormal increased activation in frontal–parietal regions evoked during WM in long-term abstinent controls (17, 19, 20, 65). Interestingly, the frontal–parietal brain regions whose activations were impacted by past SA in the controls are an overlap of regions previously shown to be important for successful performance on the *n*-back (66). It could be that the controls with past SA needed to recruit these frontal–parietal regions to a greater degree than the controls without past SA in order to maintain equivalent WM performance (Table 3).

The observation of a differential effect of past SA between the controls and schizophrenia patients is consistent with some previous literature. Two neurocognitive studies also found a differential effect of past SA when comparing schizophrenia and controls with and without past SA (31, 32). These studies observed no deleterious impact of past SA in the schizophrenia groups with and without past SA, but did find that past SA was related to poorer neurocognition in the controls with past SA compared to controls without past SA. In addition, Rentzsch and colleagues (40) observed significant abnormal inhibitory brain functioning, measured by EEG, related to past cannabis abuse only in the control group, while differences were not observed between schizophrenia patients with and without past cannabis abuse.

Further regarding our findings showing schizophrenia patients with past SA did not have a higher degree of WM impairment compared to patients without past SA; it has been proposed that schizophrenia patients with past SA have less neurocognitive impairments. This may be due to that fact that these patients were less vulnerable to developing psychosis prior to SA (24). Others have suggested that schizophrenia patients with past SA may have higher levels of cognitive functioning because some degree of social skills and intact cognitive function are required to obtain and maintain an addiction (2, 3, 67). In addition, schizophrenia patients with past SA, compared to patients without past SA, tend to show significantly higher levels of life satisfaction and executive function skills related to better social functioning (68, 69), which has not been observed in controls with past SA compared to controls without past SA (70–72). Other recent fMRI studies have also provided findings that patients with past SA may have more intact neurobiological functioning during cognitive performance compared to patients without past SA. Unlike patients without past SA, patients with past SA have been shown to activate the medial prefrontal cortex during social emotion processing (33) and affect regulation (34). Løberg and colleagues (73) reported that patients with past cannabis abuse showed increased activation in the effort mode network and decreased activation in the default mode network during neurocognitive performance compared to patients without past cannabis abuse. Similar to the decreased activation differences observed between schizophrenia with and without past SA in this study, Potvin et al. (74) also showed that patients without previous cannabis abuse had decreased activation in the left superior parietal gyrus during cognitive performance relative to patients with previous cannabis abuse and healthy controls. Indeed, it may be our results are reflecting subtly less WM-related impairments in brain activation in schizophrenia patients with past SA because of the necessity to have some level of executive function to have previously obtained and maintained their SA, though this should not in any way be construed as an endorsement of substance use.

There are limitations that need to be considered in this initial investigation. The first is the inclusion of participants with histories of abusing different types of substances. Different substances may affect neurocognitive and brain functioning differently in schizophrenia (3). An important direction for future examinations of past SA is inclusion of samples of schizophrenia and control participants with homogenous SA histories, though a much larger sample size would be needed for this. A second limitation is examining the effect of past SA on only one domain of neurocognition. Despite having the advantage of replicating across two versions of the same WM task, these results should be interpreted with some caution as other domains of neurocognition (e.g., attention, learning, and other types of memory) may be impacted differently by past SA in schizophrenia. A third limitation is the cross-sectional nature of this investigation. Future research will benefit from longitudinal studies of SA in long-term abstinent patients by more accurately capturing the trajectory of past SA in schizophrenia, and if less impaired neurocognition is static as abstinence time increases. Collecting information on family history of SA and dosage of SA could also bolster future longitudinal studies examining the relationship between SA and neurocognitive impairment in schizophrenia. Finally, because we did not do urine or hair screening for presence of substances, our results may be confounded by the reliance on self-report of the absence of current SA.

In summary, we found a differential impact of past SA on WM-related brain function in controls vs. schizophrenia patients. Controls with past SA displayed increased WM-related brain activation in several frontal-parietal-thalamic regions relative to controls without past SA. In contrast, schizophrenia patients with and without past SA only displayed differences in decreased activation in the middle frontal gyrus and inferior parietal lobule during only the *n*-back version A. These differential results are in the context of equivalent behavioral WM accuracy between control and schizophrenia patients with and without past SA. These findings are consistent with the suggestion in the literature that individuals with schizophrenia who abuse substances may have fewer neurocognitive deficits (at least initially) due to social skills involved in addiction maintenance (2, 67) and/or had less of a vulnerability to develop psychosis prior to the introduction of abusive substances (24). These preliminary findings of a differential impact of past SA on WM brain functioning among the controls and schizophrenia patients with and without past SA has important implications for the continued understanding of neurobiological processes underlying neurocognitive impairment in schizophrenia patients with and without comorbid SA.

## Conflict of Interest Statement

The authors declare that the research was conducted in the absence of any commercial or financial relationships that could be construed as a potential conflict of interest.
